# GPRC5A: An emerging prognostic biomarker for predicting malignancy of Pancreatic Cancer based on bioinformatics analysis

**DOI:** 10.7150/jca.52578

**Published:** 2021-02-02

**Authors:** Xuetian Qian, Chengfei Jiang, Shanshan Shen, Xiaoping Zou

**Affiliations:** 1 Nanjing Drum Tower Hospital Clinical College of Nanjing Medical University, Nanjing, 210008, People's Republic of China.; 2 Department of Gastroenterology, The Affiliated Drum Tower Hospital of Nanjing University Medical School, Nanjing, 210008, People's Republic of China.

**Keywords:** GPRC5A, pancreatic cancer, metastasis, epithelial-mesenchymal transition, neuroactive ligand-receptor interaction

## Abstract

**Background:** Pancreatic cancer (PaCa) is a highly lethal malignancy. The treatment options for PaCa lack efficacy. The study aimed to explore the molecular biomarkers for predicting survival of PaCa and identify the potential carcinogenic mechanisms of the selected gene.

**Methods:** Based on public databases of PaCa, differentially expressed genes (DEGs) were identified using Networkanalyst. Survival analyses were exerted on GEPIA. Oncomine and The Human Protein Atlas were used for verifying the expression on mRNA and protein levels. Enrichment analyses were generated on Metascape and gene set enrichment analysis (GSEA). Univariate analyses were performed to determine the clinical factors associated with the expression of GPRC5A.

**Results:** GPRC5A was identified as the most valuable gene in predicting survival of PaCa patients. Patients with high expression of GPRC5A showed larger tumor size, higher TNM stages, higher tumor grade, and more positive resection margin. In mutant KRAS, TP53, CDKN2A and SMAD4 group, the expression of GPRC5A was higher than non-mutant group. Mechanistically, GPRC5A may promote metastasis of PaCa mainly via regulating epithelial-mesenchymal transition (EMT) and neuroactive ligand-receptor interaction.

**Conclusion:** GPRC5A may act as an oncogene in the progression of PaCa and could be a prognostic biomarker in predicting survival of PaCa.

## Introduction

Pancreatic cancer (PaCa) is a highly lethal malignancy, with a 5-year survival rate less than 9% 1. It has been predicted to rank the second cause of cancer-associated mortality by 2030 2. Surgical resection is the dominated treatment for PaCa, yet the recurrence was observed in 72% of patients after operation 3. Early metastasis is a key characteristic of PaCa, which in part leads to tumor recurrence and makes most patients lose surgical opportunities 4. In pathological issue, PaCa is characterized by apparent tumor desmoplasia, reduction in intra-tumoral vessel density and neural remodelling 5. Thus, PaCa shows resistance to radiotherapy, chemotherapy, and molecular targeted therapy. The emerging immunotherapy also demonstrates little efficacy against PaCa 6. On the other hand, PaCa is a molecularly diverse disease, driven by aberrations in at least 10 core signaling pathways 7. Currently, molecular subtyping seems to be a new option for redefining the biology of PaCa and advancing therapeutic development 8.

To date, multiple microarray and sequencing datasets of PaCa are able to access in public. These datasets can be applied to screen genetic changes in tumorigenesis and identify potential genes with prognostic values. Meanwhile, the abundant information of the datasets has not been explored in detail, especially “The Cancer Genome Atlas” (TCGA), which contains millions of data of mutations, RNA sequencing (RNA-Seq), clinical information, copy number alteration and methylation. Bioinformatic mining methods are evolving rapidly in recent years, which make it possible for globally observing and deeply understanding the tumor biology in molecular level. Indeed, applying bioinformatic methods to deeply analyze datasets of PaCa would be beneficial for identifying the key genes underlined occurrence and progression of PaCa.

In the present study, we analyzed the differentially expressed genes (DEGs) patterns between PaCa tissue and normal pancreas, together with conducted survival analyses of the DEGs. Consequently, GPRC5A was identified as the most valuable gene in predicting survival of PaCa patients. Patients with high expression of GPRC5A showed larger tumor size, higher Tumor Node Metastasis (TNM) stages, higher tumor grade, and more positive resection margin. Mechanistically, GPRC5A may act as an oncogene in the progression of PaCa mainly via regulating epithelial-mesenchymal transition (EMT) and neuroactive ligand-receptor interaction. In summary, we preliminarily elucidated that GPRC5A could promote proliferation and metastasis of PaCa, and further validated work is needed.

## Materials and Methods

### Data collection and DEGs extraction

The selection criteria for datasets related to PaCa were as follows: 1. gene expression analysis was performed using the microarray technology or RNA-Seq technique; 2. human samples were tested. The datasets, i.e., GSE15471, GSE16515 and GSE101448, were downloaded from Gene Expression Omnibus (GEO) (http://www.ncbi.nlm.nih.gov/geo/) 9-16. NetworkAnalyst (http://www.networkanalyst.ca) was used to identify DEGs of GSE15471, GSE16515 and GSE101448 and GEPIA databases (http://gepia.cancer-pku.cn/index.html) were utilized for extracting DEGs from The Cancer Genome Atlas (TCGA) plus Genotype Tissue Expression (GTEx) datasets between PaCa and normal samples 17-22. The criteria for DEGs were adjusted *P*-value (adj. *P*) <0.01 and |log Fold Change| (|log FC|)>2.0. A comprehensive assessment of common DEGs was then performed.

### Kaplan-Meier survival analysis

The overall survival (OS) and disease-free survival (DFS) curves of the previously identified common DEGs in PaCa were generated by GEPIA under the quartile group cut-off. Statistically significant difference was considered when a log-rank *P* value < 0.05 17. The genes were selected for further analysis when both of the log-rank *P* values of OS and DFS were <0.05.

### GPRC5A expression in mRNA and protein level

Five datasets (Ishikawa Pancreas, Iacobuzio-Donahue Pancreas 2, Segara Pancreas, Grutzmann Pancreas and Buchholz Pancreas) derived from Oncomine database (https://www.oncomine.org) were used to verify GPRC5A mRNA expression 23-28. The protein expression of GPRC5A in PaCa and normal samples was verified by the immunohistochemical (IHC) result from The Human Protein Atlas (https://www.proteinatlas.org) 29.

### GPRC5A mRNA expression under different clinicopathological variables and mutant genes

The relative expression of GPRC5A across tumor and normal samples, as well as in various tumor sub-groups based on individual cancer stages, tumor grade, gender, race, age, chronic pancreatitis status, KRAS mutant status, TP53 mutant status, CDKN2A mutant status and SMAD4 mutant status was analyzed based on TCGA plus GTEx datasets 30.

### Functional enrichment analyses of DEGs between GPRC5A high-expression and low-expression group

The raw expression data was downloaded from TCGA database including 179 PaCa samples. The samples from metastatic tissues or of which the histological diagnosis discrepancy with PaCa were excluded. 177 PaCa samples were included for further analysis. Depending on the expression of GPRC5A, patients were divided into 2 groups: the top 25% (high expression, n=45) and the last 25% of patients (low expression, n=45). NetworkAnalyst (http://www.networkanalyst.ca) was utilized to screen DEGs between GPRC5A high-expression and low-expression groups. Genes with adj. *P* <0.05 and |log FC|)>1.0 were considered as DEGs.

Biological interpretation of the screened DEGs was finished by computational functional analyses on Metascape (http://metascape.org) 31, which is an online analytical tool based on several bioinformatics resources (Gene Ontology (GO), Kyoto Encyclopedia of Genes and Genomes (KEGG) and Reactome).

### Gene set enrichment analysis (GSEA)

GSEA software was exerted between GPRC5A high-expression and low-expression groups to analyze the enrichment results 32. The gene sets with nominal *P* <0.05, |normalized enrichment score| (|NES|)>1.0 and false discovery rate (FDR)<25% were considered as statistically significant.

### Association between clinical characteristics and GPRC5A

The clinical characteristic data were derived from TCGA database. Univariate analyses were performed to determine the clinical factors associated with mRNA expression of GPRC5A.

### Statistical analysis

IBM SPSS V.23 Software (Statistical Package for the Social Sciences, IBM, New York, USA) was used for the statistical analysis. χ^2^ or Fisher's exact tests were used to compare distribution of categorical factors between the different groups. An unpaired Student t test or a Mann-Whitney test was used for comparisons of two groups. *P* <0.05 was considered statistically significant.

## Results

### High expression of GPRC5A was associated with a poor DFS and OS in PaCa

**Figure 1:**
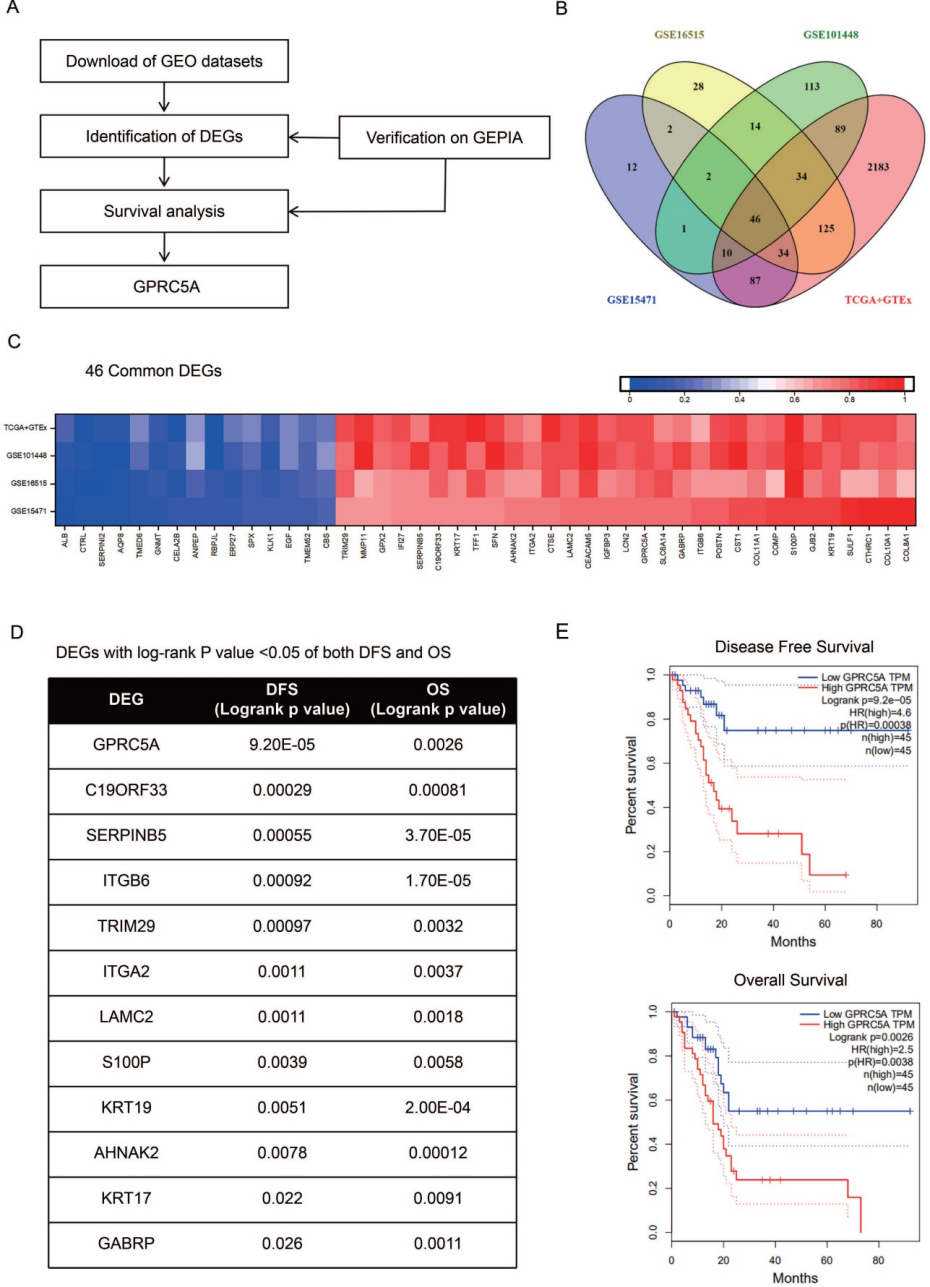
** Identification of GPRC5A as a significant gene with prognostic value in PaCa.** (A) Flow chart of selection. (B) 46 common DEGs between GSE15471, GSE16515, GSE101448 and TCGA plus GTEx datasets. (C) Log FC values of 46 common DEGs in GSE15471, GSE16515, GSE101448 and TCGA plus GTEx datasets. The red grids represent up-regulated genes and the blue grids represent down-regulated genes. (D) The genes with log rank* P* value <0.05 of both DFS and OS. (E) The DFS and OS Kaplan-Meier curves of GPRC5A. The red curve represents GPRC5A high-expression group and the blue curve represents GPRC5A low-expression group.

As the procedure shown in Fig. 1A, we extracted DEGs from GSE15471, GSE16515, GSE101448 and TCGA plus GTEx datasets and exerted survival analyses on the common DEGs. Based on the survival results, we picked the gene with the most significant prognostic value for further analysis. 213, 285 and 309 genes respectively from GSE15471, GSE16515, and GSE101448 datasets were identified as DEGs based on |log FC|>2.0 and *P* < 0.01. 2608 DEGs were extracted from TCGA plus GTEx datasets based on the same criteria above via another online tool GEPIA. 46 DEGs were marked as common changed genes between the four datasets, including 31 up-regulated DEGs (TRIM29, MMP11, GPX2, IFI27, SERPINB5, C19ORF33, KRT17, TFF1, SFN, AHNAK2, ITGA2, CTSE, LAMC2, CEACAM5, IGFBP3, LCN2, GPRC5A, SLC6A14, GABRP, ITGB6, POSTN, CST1, COL11A1, COMP, S100P, GJB2, KRT19, SULF1, CTHRC1, COL10A1 and COL8A1) and 15 down-regulated DEGs (ALB, CTRL, SERPINI2, AQP8, TMED6, NMT, CELA2B, ANPEP, RBPJL, ERP27, SPX, KLK1, EGF, TMEM52 and CBS) (Fig. 1B-C). The values of log FC- cancer vs. normal, were shown in Fig. 1C.

The survival information of the 46 common DEGs were analyzed on GEPIA. Of them, 12 genes were meaningful in predicting overall survival and disease free survival, namely GPRC5A, C19ORF33, SERPINB5, ITGB6, TRIM29, ITGA2, LAMC2, S100P, KRT19, AHNAK2, KRT17 and GABRP (Fig. 1D). Among them, GPRC5A and SERPINB5 had the most significant value in DFS and OS, respectively (GPRC5A in DFS: *P*=9.20E-05; SERPINB5 in OS: *P*=3.70E-05). In addition, ITGB6 and KRT19 also showed great values in predicting poor OS of PaCa (ITGB6 in OS: *P*=1.70E-05; KRT19 in OS: *P*=2.00E-04). Of note, high expression of GPRC5A was both correlated with a poor DFS (log-rank *P*=9.20E-05, HR=4.6) and OS (log-rank *P*=0.0026, HR=2.5) (Fig. 1E). G protein-coupled receptor class C group 5 member A (GPRC5A), also known as retinoic acid-induced gene 3 (RAI3) or retinoic acid-induced gene 1 (RAIG1) is a member of class C orphan G protein-coupled receptors (GPCRs) 33. Recently, studies have reported that it could promote malignancy of PaCa 34-36.

### Higher mRNA and protein expression of GPRC5A in PaCa compared with normal pancreas tissues

**Figure 2:**
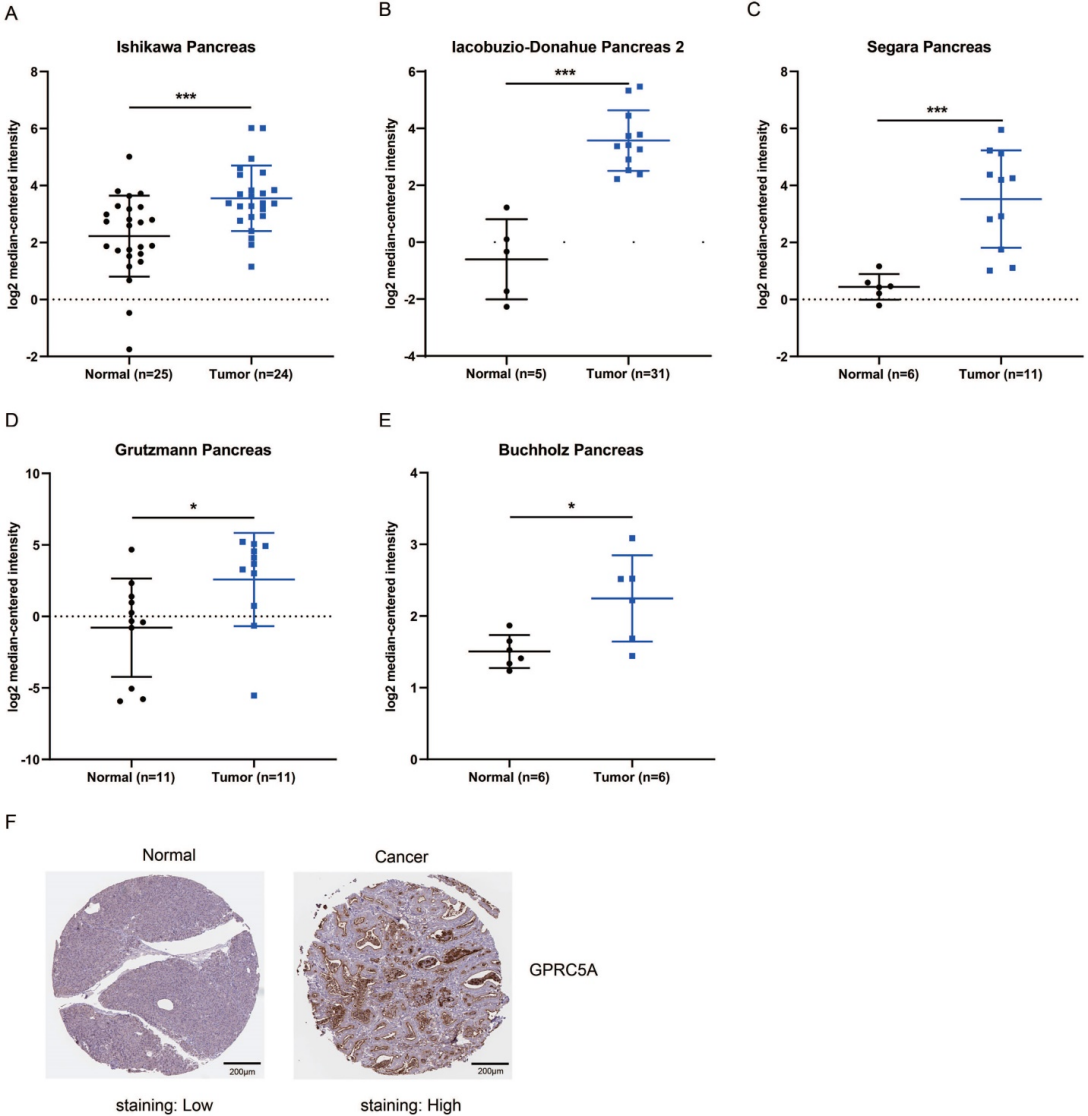
** GPRC5A mRNA and protein expression were both significantly higher in PaCa than in normal pancreas tissues.** (A-E) Scatter plots showing GPRC5A mRNA expression in, respectively, Ishikawa Pancreas (A), Iacobuzio-Donahue Pancreas 2 (B), Segara Pancreas (C), Grutzmann Pancreas (D) and Buchholz Pancreas (E). (F) Representative immunohistochemistry images of GPRC5A in PaCa and normal pancreas tissues (The Human Protein Atlas), scale: 200 µm. Normal image URL: https://images.proteinatlas.org/7928/155465_A_2_3.jpg; Cancer image URL: https://www.proteinatlas.org/ENSG00000013588-GPRC5A/pathology/pancreatic+cancer#img.

Next, we validated the expression level of GPRC5A in tumors and normal tissues of PaCa in more datasets based on Oncomine and The Human Protein Atlas. As shown in Fig. 2A-E, in Ishikawa, Segara, Iacobuzio-Donahue, Grutzmann and Buchholz studies, GPRC5A was ranked in top 1%, 2%, 4%, 4% and 11% over-expression genes with the fold change of 2.505, 8.452, 18.047, 10.324 and 1.671, respectively. Besides, the protein expression of GPRC5A in tumor was higher than in normal pancreas validated via IHC on the online tool The Human Protein Atlas. Typical images of the IHC results were shown in Fig. 2F.

### GPRC5A transcription in subgroups of patients with PaCa

**Figure 3:**
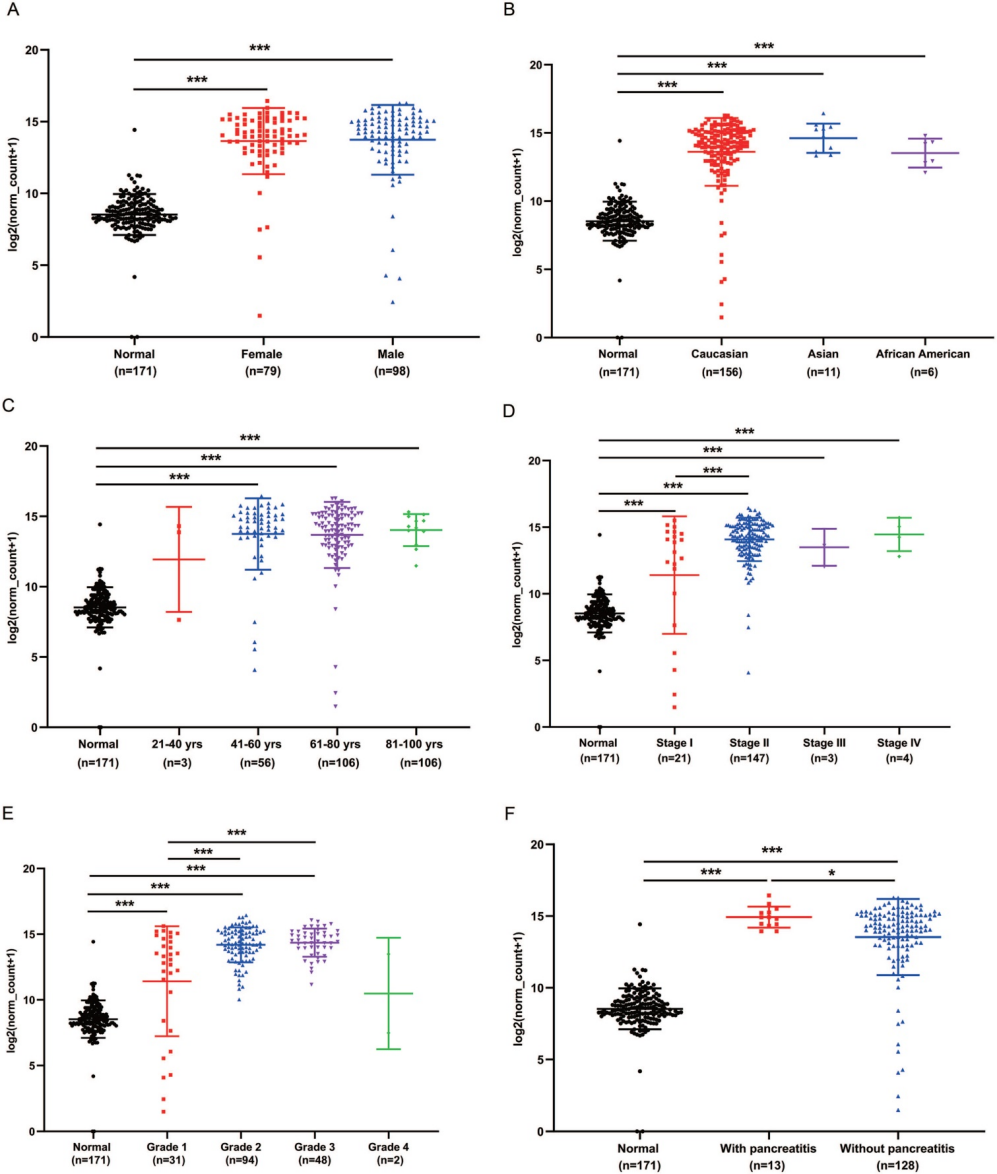
** GPRC5A transcription in subgroups of patients with PaCa, stratified based on gender, race, age and TNM stages, grade and with or without pancreatitis.** (A) Scatter plot showing expression of GPRC5A in normal individuals of either gender or male or female PaCa patients. (B) Scatter plot showing expression of GPRC5A in normal individuals of any ethnicity or in PaCa patients of Caucasian, African-American or Asian ethnicity. (C) Scatter plot showing expression of GPRC5A in normal individuals of any age or in PaCa patients aged 21-40, 41-60, 61-80, or 8-100 yrs. (D) Scatter plot showing expression of GPRC5A in normal individuals or in PaCa patients in TNM stages 1, 2, 3 or 4. (E) Scatter plot showing expression of GPRC5A in normal individuals or PaCa patients with grade 1, 2, 3 or 4 tumors. (F) Scatter plot showing expression of GPRC5A in normal individuals or PaCa patients with or without pancreatitis. Data are mean ± SD. *, *P* < 0.05; **,* P* < 0.01; ***, *P* < 0.001.

We analyzed the expression of GPRC5A in different subgroups of PaCa to see whether GPRC5A was correlated with some typical factor. Compared with the normal group, both female and male patients had significantly higher expression of GPRC5A (*P*<0.001). Between the female and male patient group, no statistical difference was shown (Fig. 3A). In Fig. 3B, patients in all races, i.e., Caucasian, Asian and African American, showed higher GPRC5A expression than normal (*P*<0.001). Same as above, there was no statistical difference between the races. In terms of age, except the group of 21-40 yrs, patients in the other three groups showed higher expression of GPRC5A than normal (*P*<0.001). Between the PaCa subgroups of age, no difference was shown (Fig. 3C). Based on TNM stages, we found that patients in each stage had higher expression than normal pancreas (*P*<0.001). Besides, the expression of GPRC5A in Stage II was significantly higher than Stage I (*P*<0.001). An increasing trend of GPRC5A expression was observed from Stage I to Stage IV (Fig. 3D). As shown in Fig. 3E, in the tumor grade subgroups, the GPRC5A transcription of grade1-3 was all higher than the normal group. Both in PaCa patients with pancreatitis or without pancreatitis, GPRC5A expression was higher than normal pancreas. Of note, between the two groups, patients with pancreatitis showed higher GPRC5A expression than those without pancreatitis (*P*<0.05) (Fig. 3F).

### PaCa patients with mutant KRAS, TP53, CDKN2A or SMAD4 had higher expression of GPRC5A

**Figure 4:**
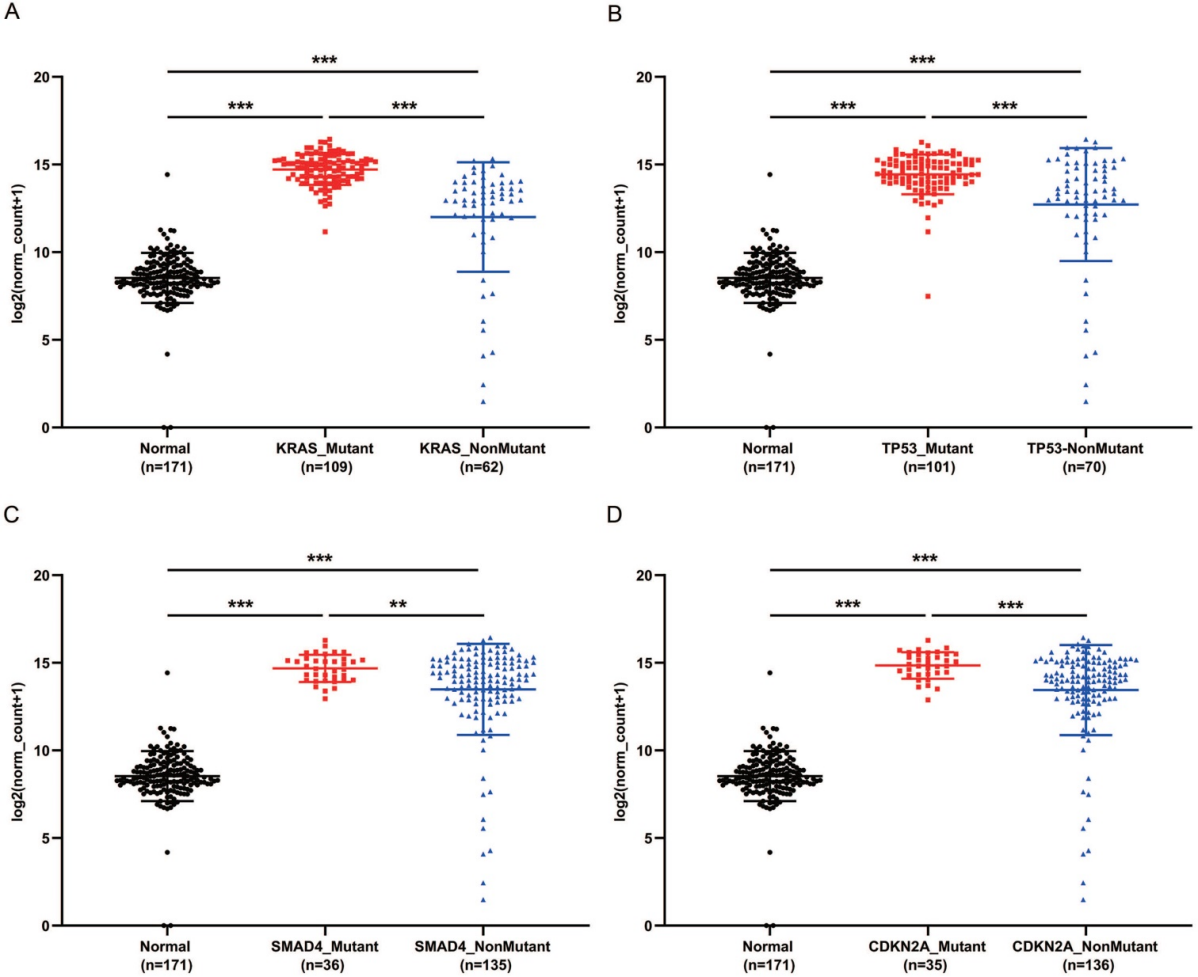
** GPRC5A transcription in subgroups of patients with pancreatic cancer, stratified based on KRAS, TP53, SMAD4 and CDKN2A mutation.** (A-D) Scatter plot showing expression of GPRC5A in normal individuals or PaCa patients with or without mutant KRAS (A), TP53 (B), SMAD4 (C) and CDKN2A (D). Data are mean ± SD. *, *P* < 0.05; **, *P* < 0.01; ***, *P* < 0.001.

KRAS, TP53, CDKN2A and SMAD4 are the most common mutant genes in PaCa 7. Regardless of mutation or non-mutation of the four genes, GPRC5A expression of tumor tissues was significantly higher than normal pancreas (*P*<0.001). Of interest, in either mutant KRAS, TP53, CDKN2A or SMAD4, the expression of GPRC5A was much higher than the group with the corresponding non-mutation group (Fig. 4A-D). The PaCa individuals with extremely low expression of GPRC5A, which was smaller than the median expression of normal group, oddly gathered in all non-mutation groups.

### Enrichment analyses of DEGs between GPRC5A high and low expression groups

**Figure 5:**
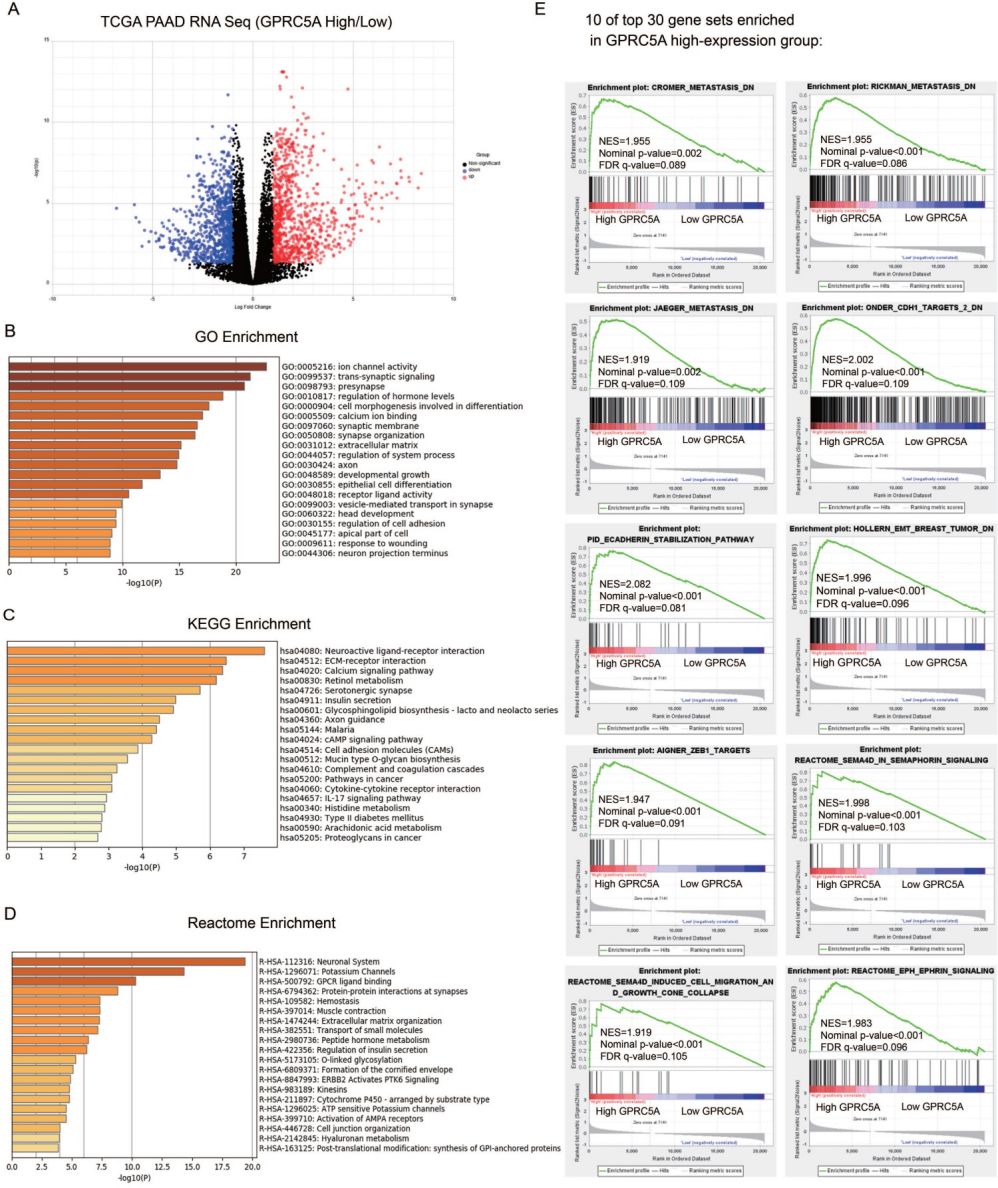
** Identification of DEGs between GPRC5A high-expression and low-expression groups, and enrichment analyses of DEGs.** (A) Volcano plot of all DEGs between GPRC5A high-expression and low-expression group. Up-regulated genes, down-regulated genes and unchanged genes were marked as red, blue and gray dots, respectively. (B) Top 20 terms of GO enrichment analysis of DEGs. (C) Top 20 pathways of KEGG enrichment analysis of DEGs. (D) Top 20 terms of enrichment analysis in Reactome Gene Sets. (E) 10 of top 30 gene sets enriched in GPRC5A high-expression group analyzed by GSEA.

According to the expression of GPRC5A, we divided PaCa patients of TCGA cohort into high-expression and low-expression group. As demonstrated in Fig. 5A, 1806 genes were identified as DEGs between GPRC5A high and low expression groups, based on |log FC|>1.0 and *P* < 0.05. Among them, 935 genes were up-regulated and 871 genes were down-regulated.

GO, KEGG and Reactome enrichment analyses were performed by Metascape. Top 20 of the most enriched terms in the three datasets were listed respectively in Fig. 5B-D and detailed information was shown in Table S1-3. The DEGs were mainly enriched in ion channel activity, trans-synaptic signaling, presynapse, regulation of hormone levels and cell morphogenesis involved in differentiation by GO analysis (Fig. 5B). For the KEGG group, neuroactive ligand-receptor interaction, ECM-receptor interaction, calcium signaling pathway, retinol metabolism and serotonergic synapse were the most enriched pathways (Fig. 5C). Reactome analysis indicated that the DEGs were remarkably enriched in neuronal system, potassium channels, GPCR ligand binding, protein-protein interactions at synapses and hemostasis (Fig. 5D). Taken together, the gene sets related to neuronal system were the most mentioned and GPRC5A could play an important role in regulating nerve-related proteins.

The GSEA outcomes demonstrated that high GPRC5A expression was significantly correlated with metastasis, EMT, Semaphorin-4D (SEMA4D) in semaphorin signaling, SEMA4D induced cell migration and growth cone collapse and ephrin signaling (Fig. 5E). The other gene sets associated with tumor ranked in top 30 gene sets included hypoxia, tumor environment, NF-kB targets, RHO pathway, EGF response and epithelial differentiation (Fig. S1).

### Top 30 DEGs analyzed by GSEA between GPRC5A high and low expression groups

A ranked gene list was generated by GSEA software. The top 15 genes with positive scores were GJB3, OSBPL3, LMO7, ASAP2, RHOF, JUP, KRT19, PITX1, LGALS3, MACC1, ACSL5, MAL2, VILL, ANXA2 and SFTA2. The top 15 genes with negative scores were SESN1, SMAD4, CDIP1, RFESD, SRR, ELAC1, HSF2, PRKN, HELQ, KLHL22, ZBTB40, RFXAP, SELENOP, MAP2K4 and ELP5. Gene full name, fold change, location and protein function were listed in Table 1.

### Clinical characteristics related to GPRC5A expression

As demonstrated in Table 2, significant differences were observed between the high-expression and low-expression of GPRC5A patients regarding size of tumor (T), TNM stage, grade (G) and resection status (all *P* < 0.05). On the aspect of size, 41 patients (91.1%) with high-expression of GPRC5A were grouped into T3/4, whereas only 29 (67.4%) patients were T3/4 in low-expression of GPRC5A group (*P*=0.014). Furthermore, patients with high-expression of GPRC5A showed higher TNM stages (*P*=0.015). In terms of grade, 40 patients (88.9%) with high-expression of GPRC5A were in Grade 2/3, which displayed significantly poorer differentiation than GPRC5A low-expression group (*P*=0.011). Besides, 17 patients (41.5%) showed positive resection margin in GPRC5A high-expression group, while only 8 patients (18.2%) were positive resection margin in low-expression group (*P*=0.038). However, in terms of age, gender, lymph node and metastasis, no statistical differences were found between the two groups.

## Discussion

Pancreatic cancer is highly aggressive and malignant, lacking efficacy molecular targets. Herein, we compared the gene expression of PaCa tissue and normal pancreas and identified 31 up-regulated DEGs and 15 down-regulated DEGs among four datasets. Further analysis revealed that 12 genes showed prognostic values, among which GPRC5A had the most significant prognostic value in DFS. The PaCa patients with higher expression of GPRC5A had a worse prognosis in both DFS and OS. In mutant KRAS, TP53, CDKN2A or SMAD4 group, GPRC5A expression was significantly higher than non-mutant group. Enrichment analysis revealed that GPRC5A could play a major role in metastasis of PaCa via regulating neuroactive ligand-receptor interaction and EMT. The analysis of clinical characteristics indicated that patients with high-expression of GPRC5A had larger tumor size, higher TNM stages, higher tumor grade, and more positive resection margin.

### GPRC5A acted as an oncogene in PaCa

G protein-coupled receptor class C group 5 member A (GPRC5A), also known as retinoic acid-induced gene 3 (RAI3) or retinoic acid-induced gene 1 (RAIG1) is a member of class C orphan GPCRs, located on the cell membrane and endoplasmic reticulum 33, 37. The current studies confirm that GPRC5A has a retinoic acid response element (RARE) in its 5' upstream region and the transcription of the gene can be induced by retinoic acid 38, 39. In addition, cAMP, TP53, BRCA1 and hypoxia-inducible factors (HIFs) were reported to regulate the transcription of GPRC5A 40-43. The expression of GPRC5A differed in various human cancers, indicating its dual role in tumorigenesis. In colorectal cancer study, GPRC5A was induced by hypoxia and was regulated major by HIFs. The downstream activation of YAP by hypoxia required GPRC5A, which enabled hypoxic cell survival by suppressing apoptosis via BCL-XL induction 40. Studies in gastric cancer also suggested that GPRC5A was an oncogene exerting its function by regulating EMT or the epidermal growth factor receptor (EGFR) signaling 44, 45. Sawada et al. reported that in prostate cancer GPRC5A facilitated cell proliferation through cell cycle regulation and was significantly essential for bone metastasis 46. Besides, in ovarian cancer, it was shown that the adaptive ERK1/2-RSK1/2-EphA2-GPRC5A signaling switch triggered chemotherapy resistance and identified GPRC5A as a marker for abysmal ovarian cancer outcome 47. In contrast, GPRC5A is generally recognized as a tumor suppressor gene in lung cancer 48. Deletion of Gprc5a resulted in persistent signal transducer and activator of transcription 3 (Stat3) activation that was important for lung cancer cell survival and transformation 49.

In the present study, we identified that in both mRNA and protein level, GPRC5A was lowly expressed in normal pancreas while significantly up-regulated in PaCa tissues. We also found that high-level GPRC5A usually predicted significantly shorter OS and DFS (see Fig. 1). As shown in Table 2, high expression of GPRC5A was associated with worse clinical characteristics among PaCa patients. Recently, Zhou et al. reported that GPRC5A promoted PaCa cell growth and migration, and enhanced resistance to gemcitabine by regulating human antigen R (HuR), which was a key mediator of gemcitabine's efficacy in cancer cells 36. Liu et al. verified the cancer-promoting role of GPRC5A in PaCa and found that when GPRC5A was knocked out, the inactivated phosphorylation of GSK-3β (Glycogen synthase kinase-3β) was upregulated 35. Jahny et al. also found that GPRC5A activated STAT3 in PaCa, which was discrepancy with its function on STAT3 in lung cancer 34*.* The function of GPRC5A in cancers may need to be discussed under specific context of tumor. To sum up, GPRC5A acted as an oncogene in PaCa.

### GPRC5A collaborated with driver genes in PaCa

In the present, compared with non-mutant group, GPRC5A was expressed higher in the patients with mutant KRAS, TP53, CDKN2A or SMAD4, which are the four key diver genes in PaCa 8.

Previous studies identified that in most tumor cell lines expressing mutant p53, the expression of GPRC5A was elevated, whereas it was relatively repressed in the tumor cell lines expressing wild-type p53 43. p53 interacted with the promoter of GPRC5A and repressed its expression at the onset of apoptosis 43. Recently, it was reported that Gprc5a-/- mice spontaneously developed lung adenocarcinomas with or without chronically nicotine-specific carcinogen exposure. Their mutational landscape exhibited markedly high somatic mutation burdens in the Kras oncogene 50, 51. It was also reported that depletion of Gprc5a activated TGF-β signaling in podocytes 52. So far, studies about the correlation between GPRC5A and the driver genes are still limited. In our analysis, higher expression of GPRC5A was shown in the mutant group, which indicated that GPRC5A collaborated with key driver genes in PaCa. More studies are needed to clarify the natural order and causal relationship between GPRC5A and the four mutant genes.

A number of drugs targeting GPCRs have been successfully designed, with a market composition of 20%-30% 53. Considering the role of GPRC5A as a member of GPCR of receptor class C, it has the potential to be a new target for drug discovery, although there is still no evidence supporting that it can be ligand activated 54. Up to date, studies on GPRC5A are still limited. However, with the development of screening technologies, the extreme abnormal expression of GPRC5A has been noticed in different cancers. Thus, its signaling function and identification of ligands deserve more nuanced exploration.

### GPRC5A may regulate neuroactive ligand-receptor interaction and EMT

In the study, the GO, KEGG and Reactome enrichment of DEGs focused on trans-synaptic signaling, pre-synapse, neuroactive ligand-receptor interaction and neural system, respectively. The results of GSEA also enriched in the gene sets related to axon guidance-associated signaling, i.e., Ephrin and SEMA4D signaling. Up to 100% of PaCa patients had perineural invasion 55-57. Neural plasticity is an intrinsic characteristic of pancreatic cancer, which involves neuronal activation at different levels in the nerve 58, 59. The severity of neural invasion was shown to be an independent prognostic factor for OS in PaCa 58, 60. Recent studies also demonstrated that ephrin and SEMA4D signaling promoted malignancy of PaCa 61-63. GPRC5A was reported to directly interact with ephrin type-A receptor 2 (EphA2), a receptor tyrosine kinase (RTK) which played a key role in epithelial tumor metastases and invasiveness 47, 64.

On the other hand, SEMA4D is expected to be a new target for treating PaCa 62, 63. Phase I clinical trial of a humanized anti-Sema4D antibody VX15/2503 (Vaccinex) has been shown to prolong 8 weeks of progression-free survival 62. Based on bioinformatics analysis, we first raised the possibility of the correlation between the signaling modulated by the two genes. The correlation could be expected. Recent studies showed that SEMA4D mediated activation of transforming protein RhoA (RHOA) and activation of RHOA could upregulate GPRC5A 65, 66. The relation could be linked by Rho GTPases; however, it needs more studies to verify.

Apart from pathways related to neuroactive ligand-receptor interactions, GESA results also showed that high-expression of GPRC5A was significantly associated with the gene sets of metastasis and EMT, which indicated that GPRC5A might play an important role in promoting metastasis (see in Fig. 5). Studies on GPRC5A regulating EMT are few. However, an explanation could be the activation of STAT3 mediated by TGF-β in cooperation with active K-Ras inducing EMT 67. In addition, active YAP and inactive GSK-3β may also mediate EMT 68, 69. Moreover, we also listed the other ten important gene sets enriched in high-expression group (Fig. S1). It seemed that GPRC5A could affect signaling related to hypoxia, tumor environment, Rho GTPase, NF-kB and EGF response. These enrichment results were in accordance with the clinical outcomes and supported that GPRC5A acted as an oncogene in PaCa.

## Limitation of the study

The limitation of the study was the unequal distribution of the patients enrolled in our analysis. The sample size of the group of 21-40 yrs was small, which influenced the statistical comparison. Since the patients included were gathered in Stage I and II, the numbers of patients included in Stage III and IV, or in grade 4 group, were quite small. Therefore, more samples distributed equally are needed to be involved to validate the results.

## Conclusion

Our study presented an overview of GPRC5A in PaCa by utilizing and analyzing the public datasets. The results strongly suggested that GPRC5A could exert pro-malignancy in PaCa. Besides, we also offered some new target genes and pathways related to GPRC5A, which deserved further exploration.

## Supplementary Material

Supplementary figures and tables.

## Figures and Tables

**Table 1:** Top 30 DEGs by GSEA analysis

Gene symbol	Gene name	Fold change; GPRC5A, high/low	Location	Protein function
SMAD4	SMAD family member 4	-0.91	Nucleus/cytosol	Transcription factor
RFXAP	Regulatory factor X associated protein	-0.96	Nucleus	Transcription factor
HSF2	Heat shock transcription factor 2	-0.74	Nucleus	Transcription factor
ZBTB40	Zinc finger and BTB domain containing 40	-0.68	Nucleus	Transcription factor
PITX1	Paired like homeodomain 1	4.43	Nucleus	Transcription factor
MACC1	Metastasis-associated in colon cancer protein 1	2.04	Nucleus/mitochondrion	Transcription activator
MAP2K4	Mitogen-activated protein kinase kinase 4	-0.58	Nucleus/cytosol	Kinase
RHOF	Rho in filopodia	2.65	Extracellular/cytosol/cytoskeleton	GTPase
SESN1	Sestrin 1	-1.21	Nucleus/cytosol	Enzyme
PRKN	Parkin RBR E3 ubiquitin protein ligase	-1.16	Nucleus/cytosol/mitochondrion	Enzyme
ELAC1	ElaC ribonuclease Z 1	-1.01	Nucleus/cytosol	Enzyme
SRR	Serine racemase	-0.87	Cytosol	Enzyme
HELQ	Helicase POLQ like	-0.51	Nucleus	Enzyme
ACSL5	Acyl-CoA synthetase long chain family member 5	2.12	Membrane/nucleus/mitochondrion	Enzyme
GJB3	Gap Junction Protein Beta 3	4.43	Membrane	Transporter
ANXA2	Annexin A2	1.61	Membrane/nucleus/extracellular	Transporter
CDIP1	Cell death inducing p53 target 1	-1.24	Endosome/lysosome/membrane	Other
KLHL22	Kelch like family member 22	-0.86	Cytosol	Other
RFESD	Rieske domain-containing protein	-0.83	Unknown	Other
ELP5	Elongator acetyltransferase complex subunit 5	-0.71	Nucleus/cytosol	Other
ASAP2	ArfGAP with SH3 domain, ankyrin repeat and PH domain 2	1.37	Membrane/golgi apparatus	Other
JUP	Junction plakoglobin	1.53	Membrane/nucleus	Other
LMO7	LIM domain 7	1.54	Nucleus/cytosol	Other
OSBPL3	Oxysterol binding protein like 3	1.64	Nucleus/cytosol	Other
KRT19	Keratin 19	2.89	Extracellular/cytosol/cytoskeleton	Other
LGALS3	Galectin 3	1.83	Membrane/extracellular/nucleus	Other
MAL2	Mal, T cell differentiation protein 2	1.97	Membrane	Other
VILL	Villin like	2.66	Cytosol/cytoskeleton	Other
SFTA2	Surfactant associated 2	5.12	Extracellular/golgi apparatus	Other
SELENOP	Selenoprotein P	-1.31	Extracellular	Other

**Table 2:** Clinical characteristics and comparisons of two pancreatic cancer cohorts

	GPRC5A, Low (n=45)	GPRC5A, High (n=45)	*P* value	Total
Age (years, median)	65	65	0.486*	-
**Gender**				
Male	24	27	0.523^#^	51
Female	21	18		39
**Size of Tumor (T)**				
T1	3	1	0.014*^a^*	4
T2	11	3		14
T3	28	41		69
T4	1	0		1
Unknown	2	0		2
**Lymph Node (N)**				
N0	15	9	0.130^#^	24
N1	27	34		61
Unknown	3	2		5
**Metastasis (M)**				
M0	18	22	1.000*^a^*	40
M1	1	2		3
Unknown	26	21		47
**AJCC TNM Stage**				
I	10	2	0.015*^a^*	12
II	31	41		72
III	1	0		1
IV	1	2		3
Unknown	2	0		2
**Grade (G)**				
G1	16	5	0.011*^a^*	21
G2	17	28		45
G3	9	12		21
G4	1	0		1
Unknown	2	0		2
**Resection status**				
R0	36	24	0.038*^a^*	60
R1	7	16		23
R2	1	1		2
Unknown	1	4		5

*Unpaired t test; ^#^Χ2 Test; *^a^*Fisher's exact test.

## Abbreviations

PaCa: pancreatic cancer
DEGs: differentially expressed genes
GEO: Gene Expression Omnibus
GSEA: gene set enrichment analysis
TCGA: The Cancer Genome Atlas
GPRC5A: G protein-coupled receptor class C group 5 member A
OS: overall survival
DFS: disease-free survival
GTEx: Genotype Tissue Expression
GO: Gene Ontology
KEGG: Kyoto Encyclopedia of Genes and Genomes
EMT: epithelial-mesenchymal transition
GPCRs: G protein-coupled receptors
IHC: immunohistochemical
TNM: Tumor Node Metastasis
RTK: receptor tyrosine kinase
