# Supplementary material for: GPRC5A: An emerging prognostic biomarker for predicting malignancy of Pancreatic Cancer based on bioinformatics analysis
Source: J Cancer. 2021 Feb 2;12(7):2010–22. doi: 10.7150/jca.52578 (PMC7974517; doi:10.7150/jca.52578)
Supplement: Supplementary file 1 — Supplementary figures and tables. [file jcav12p2010s1.pdf]

GPRC5A: an Emerging Prognostic Biomarker for Predicting Malignancy of Pancreatic Cancer Based on Bioinformatics Analysis

Xuetian Qian[a], Chengfei Jiang[b], Shanshan Shen[b,*], Xiaoping Zou[a,b*]

[a] Nanjing Drum Tower Hospital Clinical College of Nanjing Medical University, Nanjing, 210008, People's Republic of China

[b] Department of Gastroenterology, The Affiliated Drum Tower Hospital of Nanjing University Medical School, Nanjing, 210008, People's Republic of China

*Corresponding authors: Xiaoping Zou, Shanshan Shen

Email address: Xiaoping Zou: zouxp@nju.edu.cn; Shanshan Shen: shenshanshan@njglyy.com

Supplementary materials

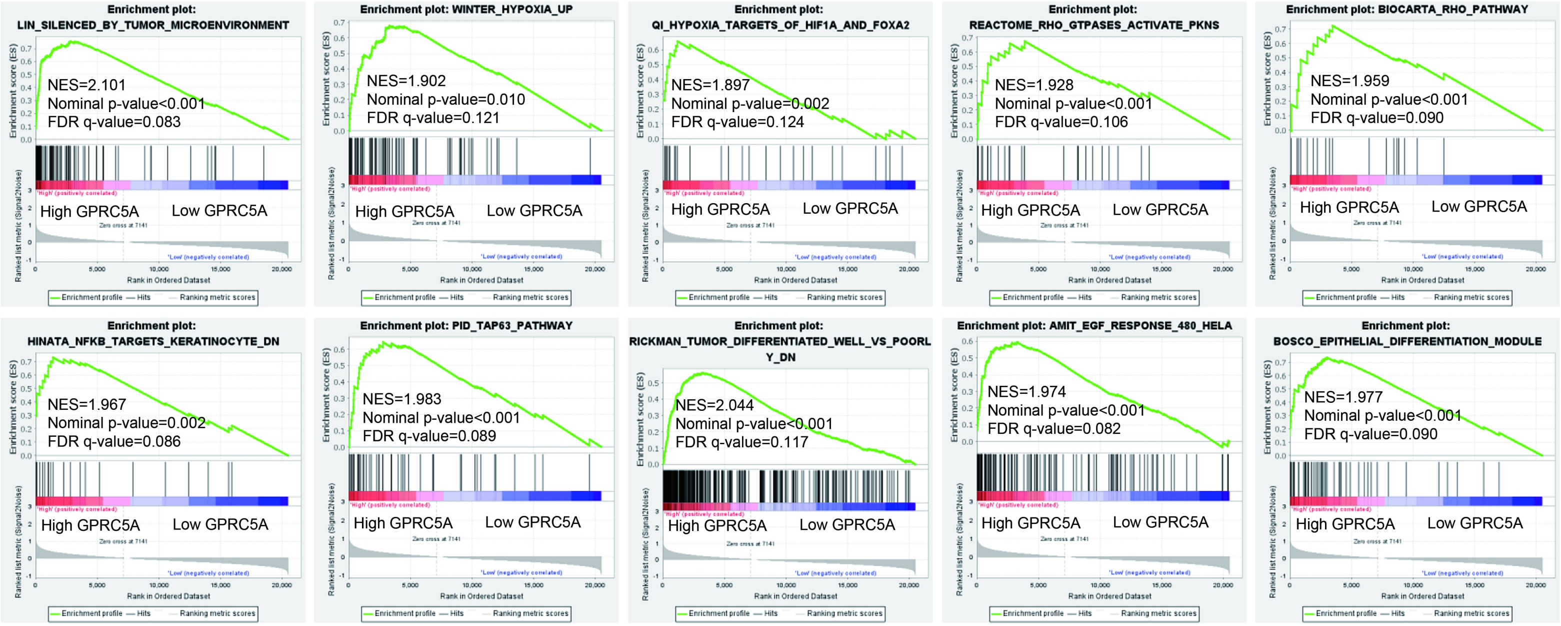

Figure S1. 10 of top 30 gene sets associated with cancer development enriched in GPRC5A high expression group.

Table S1.

Top 20 clusters with their representative enriched terms (one per cluster) in GO functional sets

GO	Category	Description	Count	%	Log10(P)	Log10(q)
GO:0005216	GO Molecular Functions	ion channel activity	94	5.23	-22.67	-18.32
GO:0099537	GO Biological Processes	trans-synaptic signaling	131	7.29	-21.23	-17.70
GO:0098793	GO Cellular Components	presynapse	105	5.85	-20.70	-17.32
GO:0010817	GO Biological Processes	regulation of hormone levels	103	5.73	-18.80	-15.73
GO:0000904	GO Biological Processes	cell morphogenesis involved in differentiation	126	7.02	-17.58	-14.59
GO:0005509	GO Molecular Functions	calcium ion binding	119	6.63	-17.05	-14.10
GO:0097060	GO Cellular Components	synaptic membrane	80	4.45	-16.59	-13.66
GO:0050808	GO Biological Processes	synapse organization	85	4.73	-16.36	-13.45
GO:0031012	GO Cellular Components	extracellular matrix	95	5.29	-15.12	-12.39
GO:0044057	GO Biological Processes	regulation of system process	104	5.79	-14.91	-12.21
GO:0030424	GO Cellular Components	axon	106	5.90	-14.80	-12.12
GO:0048589	GO Biological Processes	developmental growth	107	5.96	-13.27	-10.68

GO:0030855	GO Biological Processes	epithelial cell differentiation	114	6.35	-11.68	-9.19
GO:0048018	GO Molecular Functions	receptor ligand activity	78	4.34	-10.51	-8.09
GO:0099003	GO Biological Processes	vesicle-mediated transport in synapse	45	2.51	-9.92	-7.53
GO:0060322	GO Biological Processes	head development	107	5.96	-9.41	-7.06
GO:0030155	GO Biological Processes	regulation of cell adhesion	100	5.57	-9.38	-7.04
GO:0045177	GO Cellular Components	apical part of cell	66	3.67	-9.05	-6.72
GO:0009611	GO Biological Processes	response to wounding	96	5.35	-8.91	-6.59
GO:0044306	GO Cellular Components	neuron projection terminus	34	1.89	-8.87	-6.56

"Count" is the number of genes in DEGs with membership in the given ontology term.

"%" is the percentage of all of DEGs that are found in the given ontology term.

"Log10(P)" is the p-value in log base 10.

"Log10(q)" is the multi-test adjusted p-value in log base 10.

Table S2.

Top 20 clusters with their representative enriched terms (one per cluster) in KEGG functional sets

GO	Category	Description	Count	%	Log10(P)	Log10(q)
hsa04080	KEGG Pathway	Neuroactive ligand-receptor interaction	48	2.67	-7.61	-4.77
hsa04512	KEGG Pathway	ECM-receptor interaction	21	1.17	-6.47	-4.00
hsa04020	KEGG Pathway	Calcium signaling pathway	34	1.89	-6.36	-4.00
hsa00830	KEGG Pathway	Retinol metabolism	18	1.00	-6.18	-3.94
hsa04726	KEGG Pathway	Serotonergic synapse	24	1.34	-5.69	-3.55
hsa04911	KEGG Pathway	Insulin secretion	19	1.06	-4.97	-2.98
hsa00601	KEGG Pathway	Glycosphingolipid biosynthesis - lacto and neolacto series	10	0.56	-4.92	-2.98
hsa04360	KEGG Pathway	Axon guidance	29	1.61	-4.49	-2.73
hsa05144	KEGG Pathway	Malaria	13	0.72	-4.41	-2.69
hsa04024	KEGG Pathway	cAMP signaling pathway	31	1.73	-4.26	-2.57
hsa04514	KEGG Pathway	Cell adhesion molecules (CAMs)	24	1.34	-3.86	-2.23
hsa00512	KEGG Pathway	Mucin type O-glycan biosynthesis	9	0.50	-3.54	-1.96

hsa04610	KEGG Pathway	Complement and coagulation cascades	15	0.84	-3.23	-1.74
hsa05200	KEGG Pathway	Pathways in cancer	47	2.62	-3.09	-1.65
hsa04060	KEGG Pathway	Cytokine-cytokine receptor interaction	35	1.95	-3.09	-1.65
hsa04657	KEGG Pathway	IL-17 signaling pathway	16	0.89	-2.92	-1.53
hsa00340	KEGG Pathway	Histidine metabolism	7	0.39	-2.88	-1.50
hsa04930	KEGG Pathway	Type II diabetes mellitus	10	0.56	-2.79	-1.46
hsa00590	KEGG Pathway	Arachidonic acid metabolism	12	0.67	-2.77	-1.45
hsa05205	KEGG Pathway	Proteoglycans in cancer	27	1.50	-2.68	-1.39

"Count" is the number of genes in DEGs with membership in the given ontology term.

"%" is the percentage of all of DEGs that are found in the given ontology term.

"Log10(P)" is the p-value in log base 10.

"Log10(q)" is the multi-test adjusted p-value in log base 10.

Table S3.

Top 20 clusters with their representative enriched terms (one per cluster) in Reactome functional sets

GO	Category	Description	Count	%	Log10(P)	Log10(q)
R-HSA-112316	Reactome Gene Sets	Neuronal System	88	4.90	-19.39	-16.02
R-HSA-1296071	Reactome Gene Sets	Potassium Channels	35	1.95	-14.34	-11.27
R-HSA-500792	Reactome Gene Sets	GPCR ligand binding	76	4.23	-10.29	-7.40
R-HSA-6794362	Reactome Gene Sets	Protein-protein interactions at synapses	25	1.39	-8.81	-6.04
R-HSA-109582	Reactome Gene Sets	Hemostasis	84	4.68	-7.31	-4.82
R-HSA-397014	Reactome Gene Sets	Muscle contraction	39	2.17	-7.30	-4.82
R-HSA-1474244	Reactome Gene Sets	Extracellular matrix organization	50	2.78	-7.29	-4.82
R-HSA-382551	Reactome Gene Sets	Transport of small molecules	94	5.23	-7.16	-4.74
R-HSA-2980736	Reactome Gene Sets	Peptide hormone metabolism	22	1.22	-6.36	-4.11
R-HSA-422356	Reactome Gene Sets	Regulation of insulin secretion	20	1.11	-6.20	-3.98
R-HSA-5173105	Reactome Gene Sets	O-linked glycosylation	23	1.28	-5.29	-3.10
R-HSA-6809371	Reactome Gene Sets	Formation of the cornified envelope	25	1.39	-5.08	-2.92

R-HSA-8847993	Reactome Gene Sets	ERBB2 Activates PTK6 Signaling	7	0.39	-4.87	-2.73
R-HSA-983189	Reactome Gene Sets	Kinesins	15	0.84	-4.76	-2.66
R-HSA-211897	Reactome Gene Sets	Cytochrome P450 - arranged by substrate type	16	0.89	-4.75	-2.66
R-HSA-399710	Reactome Gene Sets	Activation of AMPA receptors	4	0.22	-4.53	-2.56
R-HSA-1296025	Reactome Gene Sets	ATP sensitive Potassium channels	4	0.22	-4.53	-2.56
R-HSA-446728	Reactome Gene Sets	Cell junction organization	18	1.00	-4.00	-2.14
R-HSA-2142845	Reactome Gene Sets	Hyaluronan metabolism	7	0.39	-3.93	-2.09
R-HSA-163125	Reactome Gene Sets	Post-translational modification: synthesis of GPI-anchored proteins	18	1.00	-3.87	-2.05

"Count" is the number of genes in DEGs with membership in the given ontology term.

"%" is the percentage of all of DEGs that are found in the given ontology term.

"Log10(P)" is the p-value in log base 10.

"Log10(q)" is the multi-test adjusted p-value in log base 10.
